# Views of People with Diabetes Regarding Their Experiences of the Facilitators and Barriers in Type 1 Diabetes Inpatient Care: An Interpretative Phenomenological Analysis

**DOI:** 10.3390/bs10080120

**Published:** 2020-07-22

**Authors:** Monica Nikitara, Costas S. Constantinou, Eleni Andreou, Evangelos Latzourakis, Marianna Diomidous

**Affiliations:** 1 Department of Life and Health Sciences/ School of Science and Engineering, University of Nicosia, Cyprus 46 Makedonitissas Avenue, P.O. Box 24005, CY-1700, Nicosia CY-2417, Cyprus; andreou.el@unic.ac.cy (E.A.); latzourakis.e@unic.ac.cy (E.L.); 2 Medical School, University of Nicosia, Cyprus 46 Makedonitissas Avenue, P.O. Box 24005, CY-1700, Nicosia CY-2417, Cyprus; constantinou.c@unic.ac.cy; 3 Nursing Department, School of Sciences, National and Kapodistrian University of Athens, Athens 10679, Greece; mdiomidi@nurs.uoa.gr

**Keywords:** type 1 diabetes, nurses, inpatient care

## Abstract

Background: The aim of this study was to comprehend how people with diabetes view their experiences of the possible barriers and facilitators in inpatient care for type 1 diabetes from non-specialized nurses. Design: An interpretative phenomenology analysis (IPA) was conducted. Methods: The sample consisted of people with type 1 diabetes 1 (*n* = 24) who use the services of the state hospitals in Cyprus. The data were collected in two phases: firstly, focus groups with people with diabetes (*n* = 2) were conducted and analysed, and then individual semi-structured interviews with people with diabetes (*n* = 12) were conducted. Results: It is evident from the findings that people with diabetes experienced several barriers in diabetes inpatient care, which is concerning since this can have adverse effects on patients’ outcomes. No facilitators were reported. Conclusion: Significant results were found in relation to the barriers to diabetes inpatient care. Crucially, the findings demonstrate that all these factors can negatively affect the quality of care of patients with diabetes, and most of these factors are related not only to diabetes care but also generally to all patients who receive inpatient care. Interestingly, no participant reported any facilitators to their care, which further affected the negative perceptions of the care received.

## 1. Introduction

Type 1 diabetes mellitus (T1DM) is a prevalent condition affecting between 21 million and 42 million people globally [1]. Onset of diabetes in childhood and adolescence is associated with numerous complications, including diabetic kidney disease, retinopathy, and peripheral neuropathy, and has a substantial impact on public health resources [2], concurrently increasing the burden on the healthcare systems [3]. Hospitalized patients commonly experience a number of complications that are associated with longer admissions, more frequent readmissions, and higher mortality [4].

Inpatient diabetes care is a growing concern because people with diabetes are more frequently admitted to hospital than those without the condition, with diabetes reported to be among the five most prevalent comorbidities in hospitalized and readmitted patients [5]. This worries governments because almost two-thirds of diabetes healthcare expenses are spent on hospitalization, with a minority of people with diabetes contributing to the majority of this cost [6]. Furthermore, since there is an increasing prevalence of diabetes in the general population, this has resulted in a steady increase in beds occupied by people with diabetes [7]. At this rate, projecting forward, it has been estimated that by 2030 more than one in four inpatients will have diabetes, while in some UK hospitals the prevalence is as high as 30%, a prevalence already exceeded in some US states [6,7,8]. It is worth noting that despite the documented frequency, most people with diabetes are hospitalized for other reasons, with diabetes-related emergencies primarily implicated in fewer than 10% of admissions [7]. Nevertheless, as has been said above, inpatients with diabetes have poorer clinical outcomes with longer lengths of stay in comparison to patients without a history of diabetes, despite the fact that they may be admitted to the hospital with the same diagnosis [9,10,11].

However, the care that people with diabetes receive is suboptimal and, despite the fact that T1DM is a chronic disease with multiple admissions to hospitals for diabetes-related or unrelated conditions [12], the impact of this type is often overlooked as focus has been on the type 2 diabetes mellitus (T2DM) epidemic [13]. Many patients with T1DM experience inpatient care complications because of inadequate care while the provision of reliable and proactive inpatient care for people with diabetes is inadequate, leading diabetes inpatients to experience poor care. This is supported by recent findings showing that hospitalized people with diabetes did not see a diabetes care team because of understaffing and lack of process [7]. This means that patients might not receive the necessary specialized support required for diabetes care, which can lead to several adverse effects during hospitalization. 

Ιt is evident from the literature that the complex nature of diabetes necessitates the involvement of nurses who have multiple roles in providing care in order to achieve effective management of the disease. There is strong evidence to show that nurses have an important role in advising patients on how to be more independent by managing their disease themselves [14,15]; nurses are involved in the management of their medications, in screening for diabetes complications, in administrative duties, and in providing patients with psychological support [16]. At the same time, evidence suggests that people with diabetes often confront fragmented care, especially from nurses [17,18]. The literature demonstrates that nurses and other healthcare professionals encounter many barriers to providing optimal diabetes care. The most important findings regarding the barriers are nurses’ lack of knowledge about diabetes, lack of resources, and lack of time [19,20]. In 2015, 31.1% of acute hospital sites had no diabetes inpatient specialist nurses and 9.2% of sites did not have any consultant time for diabetes inpatient care [21]. These factors contributed to a 38.3% rate of inpatient diabetes medication errors [21]. 

A number of initiatives have been made during the last few years in order to confront this issue, such as the establishment of nurse-led diabetic clinics, the role of diabetes specialist nurse (DSN), the creation of the diabetes educator, and nurse participation at various levels of the healthcare system, such as primary care, and several other technological advancements [22,23,24]. These initiatives have been shown to have good outcomes especially in regards to primary care and prevention control, as well as having decreased unnecessary referrals to secondary care and outpatient attendances [25]. However, it is important to note that, although many countries report these initiatives, nurses’ roles and work settings differ. For example, in Sweden and the Netherlands, almost half of the DSNs work in primary care or in integrated care, while they also have the right to prescribe for people with diabetes. In contrast, most DSNs in Ireland are working in hospitals in the secondary care role and not all of them are allowed to prescribe [25]. Therefore, the role of the specialist nurse in diabetes differs among countries, and inpatient care is provided mostly by non-specialized nurses. 

Taking into consideration the large number of people with diabetes in hospitals, it is anticipated that most nurses have sufficient capability and education about diabetes inpatient care, and that patients with diabetes can expect sufficient and high-quality care. Remarkably, previous literature found that patients often indicated inadequate experiences of inpatient care, particularly in relation to the following: lack of diabetes experience amongst hospital staff (especially nurses), unsatisfactory data, and setbacks in being released because of diabetes—particularly when diabetes was not the original reason for admission [7,18]. 

The above situation is worrying because, during the last decades, governments have taken many initiatives to support those with diabetes, especially by giving extra attention to primary care and prevention, whereas they have paid little attention to diabetes inpatient care and to people with T1DM. Furthermore, no evidence has been gathered about the barriers of inpatient care from the perspectives of people with T1DM who are the recipients of this care. Therefore, since nurses have an important role in diabetes care, it is relevant to eliminate any barriers that prevent them from providing adequate care, and to enhance any facilitators that allow them to provide the best quality of care. Taking into consideration all the above, the aim of the current study was to explore and understand the views of people with T1DM regarding the inpatient care they received from non-specialized nurses. If they expressed that the care was good, we sought to find out why they thought the care was good, and what factors facilitated that care. If they thought the care was inadequate, we wanted to understand what specifically prevented them from receiving better care. 

## 2. Materials and Methods

The current study reflects interpretivist epistemology, is informed by phenomenological social theories, and strictly follows the IPA methodology in order to better understand how people with T1DM, through their experiences, perceive the facilitators and barriers that affect the received care. In line with the theoretical underpinnings of IPA, the participant sample for this study was purposive and homogenous. More specifically, 24 people with T1DM participated in the current study. Two sources for gathering data were used—namely, focus groups and interviews. The two focus groups had six people each. Twelve individual interviews followed in order to gain a deeper understanding of the experiences of the participants. All subjects gave their informed consent for inclusion before they participated in the study. The study was conducted in accordance with the Declaration of Helsinki, and the protocol was approved by the Cyprus National Bioethics Committee (EEBK EΠ 2012 01.104). The sample covered the entire area of Cyprus, since participants were recruited from each city in Cyprus. This research followed the general principles of research ethics, consent, anonymity, and confidentiality. All the necessary approvals and licences were granted from the responsible bodies. All participants gave their informed consent for inclusion before they participated in the study.

The analysis followed the four stages suggested by Smith and Osborn [26,27], which helped to identify shared experiences across the group of participants, and in order to ensure the quality of coding the process was carried out by two researchers independently. At the 1st stage of the analysis (which Smith and Osborn named “Looking for the Themes of the First Case”), the transcripts of the first focus group were read many times and the identified themes were written down in the right-hand margin. The coding process continued in the same way with the next focus group until clusters of themes were generated. In the same way, the themes that emerged were used to formulate the interview schedule for the semi-structured interviews. After completing the semi-structured interviews, the first interview transcript was read multiple times, finalizing the cluster of themes. The themes from the first interview oriented the following analysis.

At the 2nd stage (which is the “Connecting Themes” stage), an Excel document was prepared with the themes that emerged in order to look for connections or combinations. After several examinations, some clustering themes were found and a table was prepared with the final themes. Once the results were compiled for the focus groups, the same procedure was followed as for the first interview with people with diabetes.

The “Continuing the Analysis of Other Cases” is the 3rd stage, according to Smith and Osborn (2008) [26]. Since there were two focus groups with people with diabetes, at this stage of the analysis the researcher incorporated the second focus group into the first focus group. This followed the idiographic approach to analysis, which starts with analysing particular examples and then finally arriving at more general themes [26,27]. For the purpose of the current study, we decided to use the themes that emerged from the first focus group to orient the following analysis, while we recognised new issues emerging from the following transcript. This allowed us to identify new and different themes. After analysing both transcripts, a final table with the dominant themes was developed and they were then reduced not according to their prevalence but according to their richness within the particular passages.

And at the 4th stage, the “Writing up the Results” stage, we translated the analytic themes into a narrative account. 

## 3. Quality Markers

The current research was focused on five criteria: credibility, transferability, dependability, confirmability, and reflexivity to ensure quality. We employed the triangulation method by collecting data through focus groups, and we compared the themes extracted during the focus groups and the new themes that emerged during interviews. As Constantinou et al. (2017) [28] and Vasileiou et al. (2018) [29] maintained, the saturation of the data is a main quality marker, so we used the comparative method for themes saturation (CoMeTS) to achieve themes saturation in qualitative interviews. Through this process we confirmed that there were no new data to discover, and data collection stopped. 

## 4. Results

### 4.1. Views of People with Diabetes about Facilitators and Barriers to Diabetes Care

Participants were asked to describe the factors that they perceived as facilitating nursing care or making it difficult for nurses to provide inpatient care to diabetic patients according to their lived experiences. Interestingly, none of the participants referred to anything that facilitated nurses to care for people with diabetes. 

### 4.2. Resources

People with diabetes referred to the lack of resources, which might prevent nurses from providing effective nursing care to diabetic patients. The main barrier they identified was the state of the health system, because the participants asserted that it was the government’s responsibility for the financial constraints. They believed that this caused staff shortages, and medications and other necessary consumables to be unavailable for diabetes care. The following quotes show that patients tended to highlight the importance of available resources:

In the public hospital, the resources they use are made with the cheapest materials…because the root of the problem is financial. They use the money for their cars, their limousines, their suits.

*(Focus Group 1)*

They do not have the resources to have enough staff, doctors. Financial resources.

*(Focus Group 1)*

Unfortunately, instead of choosing what would be best for the diabetic but also for every person who has a disease, they choose what is more economical for the government.

*(Focus Group 2)*

Two of the participants also referred to the government’s responsibility for the financial constraints that limited continuing education for nurses on diabetes care. More specifically, Participant 5 and Participant 1 explained:

My perception is that everything is about the money. That’s how it is in my mind. From my experience, they do not provide funding for a learning programme or exchange nurses, as they do for student exchanges.

*(Participant 5)*

Let me say that as a health ministry they cannot provide these services due to a low amount of money...I believe the health ministry should see these things and do these seminars we said earlier.

*(Participant 1)*

### 4.3. Healthcare System Barriers

Participants from both focus groups claimed that the healthcare system caused barriers for diabetes nursing care. They blamed governmental and organizational factors for the inadequate care and also indicated that there were nurses who were well-educated about diabetes care; however, bureaucratic procedures resulted in underutilising these nurses. Most of the participants who participated in one-to-one interviews also supported the above view by describing the healthcare system in Cyprus as an “unhealthy system”. The words of a participant from Focus Group 1 illustrated these findings quite well:

It is the fault of the ministry, the administration...They do not give generously to health.

*(Focus Group 1)*

Participant 10 also blamed the general healthcare system in Cyprus, but she correlated this with nurses’ lack of continuing education:

I have lost my faith in public service since I started working. In Cyprus, we have this system “I am working for the government for the rest of my life, full stop.” I assume that in all public services this is done. But I do not know if the nurses are to be blamed for this or if the presidents should be. As doctors are constantly learning, nurses need to do that as well.”

*(Participant 10)*

### 4.4. Diabetes Specialist Nurses

Some of the participants referred to the role of the diabetes specialist nurse. They recognised the importance of this role despite the fact that they did not see anyone with this specialization in the wards. Specifically, a participant from Focus Group 2 stated that there are nurses who are trained and ready to undertake such roles, however, the healthcare system does not take advantage of this resource.

*There are specialized nurses for diabetes. There are many who have attended one or two seminars. They went to the Diabetes Association and learned a lot of things. Nurses exist, they simply are not being exploited. I believe there are more than 27. Each year, 10 nurses go for training. There are enough nurses ready to work but they are lost in the process in state hospitals. If we recognized the title of diabetes specialist, then they will work on this shift in this position.*

*(Focus group 2)*

When Participant 9 was asked whether she would have accepted any information from nurses, she replied that from her experience one of the major problems with inpatient diabetes care was the lack of a diabetes specialist nurse.

*Interviewer: Would you listen to a nurse if s/he was talking to you?*

*Participant 9: Yeah, surely. But I didn’t have to, because they did not have a specialist nurse and I think that was the biggest issue.*

Participant 8 expressed a similar view in that he said he would have accepted and trusted any information provided by nurses only if they were specialized:

If he was a specialist, yes. It means he studied this, and I would have trusted him.

*(Participant 8)*

### 4.5. Information Provided to the Participants

Some of the participants expressed concerns that health professionals, including nurses, did not provide them with the necessary information.

*They should have given me an information leaflet. If I had it in writing, I would know where to go and what to be careful of, like urine, analyses, what to eat, etc.*

*(Participant 9)*

On the other hand, participants complained that the health professionals overloaded them with information regarding their treatment and the lifestyle changes that a person with diabetes needs to make. Most of them considered it as a barrier to effective diabetes management because it sometimes had the opposite effect on a patient’s adherence to the treatment.

*I want them to explain things to me, and they started: “You have to go on a diet, you have to do sports, you have to control your sugar.” It was a traumatic experience for me and I said to myself, “I don’t care: as long as I live.”*

*(Participant 4)*

The same participant reported that healthcare professionals provided conflicting information that might cause patients to become confused and to be uncertain about the care they receive.

*Because there are many opinions, but sometimes they are contradicting, and we do not know whether they are right. For example, for the insulin dose…, there are doctors who will tell you that you have to use as many units as you can, and another doctor may tell you to calculate the units based on your meal, the amount of carbohydrates, or, for example, for the treatment of hypoglycaemia, some say you have to drink juice too... While others say you have to wait for 5 min and redo the analysis.*

*(Participant 4)*

Furthermore, two of the participants from the Larnaca district referred to the repeated and negative information nurses gave them as a factor that inhibited them from following their instructions, and it was the reason for them choosing the private sector.

*They insist on some issues and they say the same thing again and again. I go to the nurse and she talks, and the next time I go, she talks again. No, I don’t go now. I was tired of hearing the nurse for four years telling me the same things.*

*(Focus Group 1)*

*Everything they say is negative. Only negative words you heard…Thus I decided to go to the private sector.*

*(Focus Group 1)*

### 4.6. Lack of Empathy

Some of the participants revealed difficulties in communicating with nurses, and this made them feel that there were misunderstandings between them. A participant from the focus group explained that the nurses did not understand her very well, and she was frustrated that nurses criticised her regarding her glucose levels and dietary habits. She explained that one day when she was at her scheduled doctor appointment, the on-duty nurse and the lab analyst were upset with her:

She also had the woman holding the analyses that will comment on your glucose because it is high, there is the nurse who will comment on why you ate that thing and your glucose is up while you’re in insulin… They do not understand… Yes, I have diabetes, yes. I have the right to eat everything, everything…

*(Focus group 1)*

The feeling that nurses did not understand them and their need to be understood was also expressed by Participant 5, who stated that currently she felt she did not have somebody to understand her, and this left her feeling insecure:

I need to feel secure. I would like to feel that someone understands me, I want to talk to someone and feel that I’m talking at the same levels… Now, I feel like I’m talking and no one understands me.

*(Participant 5)*

Another participant believed that nurses did not respect people with diabetes in relation to their disease because of their lack of knowledge about diabetes, and because they did not pay attention to their condition:

They do not respect us because they do not know. It is not something for them. It’s not something serious for them. Okay she has diabetes, we will give her white bread, she will eat it and she will not talk.

*(Focus group 1)*

### 4.7. Availability of Time

When people with diabetes were asked about factors that helped or prevented nurses from providing adequate care to their patients, some of the participants recognised that the limited amount of time available to nurses was a barrier. Patients understood that nurses were busy with overcrowded hospitals, which meant that nurses did not have enough time to pay them adequate attention and, as a result, some aspects of patient care were left incomplete. More specifically, patients said:

The people don’t understand that we all have limits, and there are too many people in the hospital, to a point that it is so full that there is no space for others.

*(Focus Group 2)*

In the public hospitals, there are so many people. Doctors are in a hurry; nurses are in a hurry, and they will not give you that importance you need.

*(Participant 1)*

Because of the pressed programme and the crowd, they were trying to explain to me what my problem was and they did not have the time to help me.

*(Participant 2)*

### 4.8. Nurses’ Interest in Diabetes

Some of the participants maintained that nurses did not have an interest in diabetes. They described nurses as relying on doctors to critically think about how to deal with diabetes cases because they did not care about it. They assumed that nurses considered diabetes a simple disease and that they preferred paying more attention to more critical cases.

*He will not go into the process to think, “This patient has 200 mg/dl sugar [glucose], so how much should he eat?” He will just call the doctor and ask him how much insulin he has to administer. So they do not care.*

*(Focus Group 1)*

*I think they see diabetes just as a disease...They are diabetics; a thousand of people have diabetes, so what? They will give more importance to another person whose illness is more severe.*

*(Participant 1)*

Furthermore, Participant 5, stated that through her inpatient experience she noticed the indifference of the nurses regarding her care and said that nurses were only involved in typical tasks, such as measuring blood glucose.

*They are typically involved only in measuring. Usually, I am admitted to the hospital because of ketoacidosis. When this happens, I vomit. When this happens, I would like someone to take the dirty vomiting bowl and bring me a new one. I saw some indifference. The indifference of the people.*

*(Participant 5)*

### 4.9. Lack of Nurses’ Autonomy

One important finding that participants mentioned was that nurses did not have the autonomy to take responsibility for their patients. Participant 8 clearly stated that:

Nurses do not take responsibility. They do not take responsibility to tell you whether you will reduce or increase your insulin.

*(Participant 8)*

Participant 10 added that it was a problem that nurses were afraid to take responsibility, and she described her friends’ experience with a nurse who did not take the responsibility to provide him with the necessary equipment for his insulin pump:

Another problem is the nurses’ fear of responsibility. It’s not my own experience, but a friend of mine who has a pump and he had to pick up some sensors from the hospital. They told him that they did not have any. My friend stayed there and pushed them to give some to him, so the nurse went down to the warehouse with my friend and they found two boxes filled with sensors. The nurse who was with him still did not give him the sensors and disagreed. In the end, the nurse had to ask three doctors to take the responsibility and give the sensors to my friend. The nurse could not make a decision on his own.

*(Participant 10)*

Participant 12 also said that nurses relied on physicians and that they did not “dare” take responsibilities on their own:

They have specific instructions to follow. They will not go into the process of thinking about doing something on their own. For example, I’ve had high levels of sugar for three days and they didn’t go to the trouble of thinking about me needing anything else. They would not dare to find a doctor and ask him or to suggest to him something else.

*(Participant 12)*

### 4.10. Focus on Physicians’ Roles

Participants experienced different emotions when they referred to nursing care. Most of the participants claimed that they trusted and relied on their physicians, while sometimes it was apparent that they undervalued nurses’ competence.

However, there was variation in participants’ views and experiences. More specifically, a participant from the focus group affirmed that diabetes care was a personal issue and that each patient knew better about his disease than health professionals. Therefore, this participant thought there was no need to pay any attention to the nurse but to pay some attention to the doctor whenever it was needed. More specifically, a patient explained,

*I do not believe that you need doctors to support you or nurses to explain to you, I will not get support either from the doctor or the nurse or anyone... after 3, 4, [or] 5 years, you have to be your doctor... I think you should not even count on the nurse. You only count on the doctor up to a point.*

*(Focus Group 1)*

Another participant from a focus group emphasised that physicians were available and trustworthy. They were there any time a patient needed them and they knew more, while the nurses relied on physicians’ orders:

But the doctor is there at all times. It happened when I was measuring my glucose level and it was 200, and I told the nurse that is only 4 o’clock, what will happen until 6 o’clock when my meal will come? And the nurse did not know, so she called the doctor. They do not know how to deal with us. But the doctor is there. The doctor will tell her what she has to do.

*(Focus group 1)*

The above views were further supported by participants who had one-to-one interviews. They said that they trusted their physicians more because doctors had greater knowledge than nurses, and that nurses needed more education in order to make them feel safe. For example, when Participant 1 was asked why she trusted her physician more, she replied:

Because I know that the doctor will know more things. That is what I believe…I believe that nurses need to continue their education, especially in diseases like that, so that he can come to help me.

*(Participant 1)*

Participant 9 agreed with that opinion in saying that she would have accepted advice from a nurse but she felt she could trust her physician more.

In support, Participant 6 also said that he did not trust nurses’ knowledge specifically about pump insulin, and he preferred specialist physicians to educate him because of the greater knowledge they have.

*I believe that doctors specialized in diabetes have more knowledge in this area. Especially for the pump—I would not trust a nurse to regulate it.*

*(Participant 6)*

Participant 2 also trusted that physicians knew more and regarded them as the most appropriate professional to guide them. He believed that nurses could give him general information regarding diabetes but the responsibility for his medication clearly belonged to his physician. This opinion had been cultivated since he was a child. He viewed the nurse’s role as simply to perform the more technical actions.

*For example, the nurse might also tell me that diabetes is a way of life and that it is a pancreatic insufficiency. I don’t need a doctor to tell me this. However, as far as the issue is concerned about how to regulate my insulin units, the doctor has the responsibility because he is more qualified.*

*(Participant 2)*

*From an early age, I was told that the doctor knows better. My parents told me that the doctor is the right person to guide me. Based on this reasoning I never asked for anything from the nurses. My nurses just changed the IV fluids and asked if I felt well.*

*(Participant 2)*

The same view was shared by Participant 10, who said that, based on her friends’ experiences, she did not trust nurses in the state hospitals as they did not give the proper attention to their patients. Furthermore, she stated that she trusted physicians more because nurses’ education was limited in comparison to doctors:

I don’t trust the state hospitals ever since I was diagnosed with diabetes. I would not trust to have an operation and stay there. And from the things I hear from acquaintances and friends who have diabetes and have had to be hospitalized, I have no trust [in the hospitals]. They will not give me the attention they need to give me.

*(Participant 10)*

When she was asked if she would accept any suggestions about her medications from the nurses, she clearly stated:

Participant 10: I do not know if I think it’s right…I would not listen to the nurse but I would listen to the doctor… Nurses do not inspire me to trust them…

Interviewer: Why?

Participant 10: Because you know that the doctor who will tell you what to do studied endocrinology and it is his subject.

Participant 8 also regarded physicians as the only source for getting advice and information regarding his medications, while he did not trust nurses because they were not physicians. When he was asked whether the nurses could undertake this role, he stated that nurses did not have any responsibility to advise about their medications and this was the physician’s job.

*Interviewer: Would you accept a nurse telling you to reduce or increase your insulin?*

*Participant 8: No, I do not trust him. He is not a doctor. The nurses are doing what the doctor tells them. The nurse has another job.*

*Interviewer: Can you see any other professional other than the doctor to have a role in your diabetes care?*

*Participant 8: Nobody else. For example, now that my sugar levels are low, the doctor can tell me to reduce the insulin units. Unless the doctor tells me, no one else can.*

*Interviewer: What is the nurse’s job?*

*Participant 8: Treat the sick. Not to prescribe drugs. This is his job.*

A range of barriers was reported by our participants. Interestingly, none of the people with diabetes referred to any facilitator for diabetes care. However, a range of barriers was reported, which is of great concern, since this can have adverse effects on patients’ outcomes. In the following chapter, there will be an extensive discussion about all the results of the current study.

## 5. Discussion

Several barriers were identified by our participants in the provision of diabetes care by nurses in the hospital setting. The lack of resources was well documented in the literature in previous studies, in both developed and developing countries, and our findings add to the evidence. The findings of our study are in line with those of studies carried out previously [30,31,32]. Diabetes is a complex disease that requires specific medicines and equipment, such as insulin, pens, syringes, and pumps, and as the technology is constantly being updated, it can be seen that the lack of resources in diabetes care is still a barrier, according to studies published in both developed and developing countries. However, we consider the lack of resources to be related to the lack of time, mainly because both are factors that lead to patients missing care. For example, a study conducted by Rivaz et al. [33] found that the participants acknowledged the physical resources, such as sufficient and modern equipment in the workplace, as the facilitators of care and medical processes. Participants indicated the lack of sufficient equipment as a significant obstacle that negatively affected their work because they sometimes had to miss important care activities or they were delayed in delivering care, and this resulted in emotional pressures. Blackman et al. [34] also reported that inadequate physical resources and equipment predict missed care, while the accessibility of adequate contemporary equipment has a significant impact in enabling care delivery, decreasing stress levels, and improving patient satisfaction.

This can be considered a healthcare system issue because many countries, including Cyprus, are currently dealing with financial constraints, and our participants also indicated that it is the government’s responsibility because of the financial restrictions. Adding to this, Williams et al. [20] confirmed that lack of money for equipment affects quality of care, and they explained that the importance of “priority setting” is to decide at various system levels how to distribute limited funds to certain groups of patients and available treatments. Therefore, it is important that decision makers at all levels understand that “priority setting” and lack of resources are issues that lead to several other negative outcomes and that they address this scarcity in order to improve both nurses’ efficiency as well as patient outcomes.

Furthermore, our participants referred to the lack of diabetes specialist nurses in the belief that the development of this role could amend the situation because of their expert knowledge. However, their roles and work settings differ among countries, with some of the countries having them available only in the primary settings. Therefore, the care provided in inpatient services by non-specialized nurses who do not have adequate knowledge is still questionable since there are non-universal and announced measures for dealing with this issue. This is supported by the next concern of our participant, who referred to the misleading information provided by nurses. The provision of misleading information to patients can be from the lack of knowledge by nurses or from outdated knowledge. However, no research was found in the literature to study or support this finding.

Our participants also referred to the lack of empathy by nurses, which was well documented and explored in the literature. Empathy is a prosocial behaviour that is beneficial to others and is fundamental to ethical nursing practice [35]. However, Jefferey [36] argued that there is currently a problem in the balance between the scientific–technical and psychosocial elements of patient care, and that the reasons behind the lack compassion are the fatigue, overwork, excess demand, lack of continuity, and a failure to see patients as a fellow human beings despite their illness. In the literature, the most reported reason for lack of empathy from health professionals was burnout [35]. More specifically, studies reported that nurses who experienced burnout could not show empathy to their patients during nursing activities, and this could affect negatively the quality of care [37,38]. This was strongly related with the participants’ responses in relation to the lack of available time from nurses. Since nurses are struggling in overcrowded hospitals and do not have sufficient time to provide support to patients, this can result in nurses experiencing burnout and, consequently, in a lack of compassionate care.

Another correlated factor that can inhibit the role of the nurses in diabetes care is the “lack of autonomy” in their profession, and this was identified by several participants. Over the past 50 years, there have been several studies exploring the value of autonomy in the nursing profession. Our findings do not support the evidence in the literature, which found that approximately 60.4% of non-nursing health workers [39] versus only 6.7% of nurses [40] considered that a nurse has professional autonomy, which is alarming [39].

Autonomy allows nurses to make clinical decisions and exercise judgement about the care provided to their patients, using their own professional knowledge [41,42,43]. Nurse autonomy should be encouraged and allowed by the workplace, enabling nurses to make active decisions regardless of individual characteristics [44]. The authority to make decisions can occur at three levels: clinical, operational, and professional [41,43,45,46]. At the clinical level, nurses are allowed to make clinical decisions about the type of care they will provide to their patients [41]. Job autonomy has to do with the decisions nurses make in collaboration with the administrators. The autonomy at the professional level refers to the mutual decisions that nurses make according to specific professional practices and policies to guide them within an organization [44].

Literature on nurses’ autonomy showed evidence of job satisfaction as a result of autonomy, which is an important element of the work environment that allows nurses to have better outcomes [46,47]. Furthermore, the literature provided evidence that nurses in settings that support nursing autonomy express more satisfaction with their jobs, have lower rates of burnout, and are less likely to leave the profession. Furthermore, nurses relate their ability to make autonomous decisions with better quality of care and greater teamwork [48].

However, the literature revealed factors that limit the autonomy of the nurse in the hospital environment that are consistent with other findings of our study. These factors include: the influence of the physician on the work of the nurse; the deficiency of technical–scientific knowledge; physical and emotional exhaustion from work overload; inadequate physical structure; scarcity of material; compliance with medical orders; and the nurse’s dependence on the physician to perform some care and/or action [49]. Therefore, one could argue that the views of our participants confirmed that nurses lack autonomy, since most of these factors were also identified in our study. Furthermore, it is interesting that the lack of autonomy in our nurses can negatively impact the quality of care for patients as well as the nurses’ self-satisfaction.

Additionally, patients’ “lack of trust” in nurses was one of the most important findings that was not consistent with the literature. For instance, our participants showed they value physicians’ roles rather than nurses’, whereas in the literature, studies that estimated patients’ levels of trust in nurses in other fields, and not specifically in diabetes care, indicated that nurses are highly trusted by patients. This inconsistency between our findings and the wider literature might be due to the fact that the medical profession has a privileged position in Cyprus society, while nurses do not. This confirmed Loizous’ [50] findings that people with diabetes seem to rely to a great extent on their physicians not only for their medications but also for psychological support. This is an interesting finding. Although general trust in physicians plays a significant role in patient care, evidence in the global literature showed that public and patients’ trust toward their medical profession has seemingly reduced [51].

However, the lack of trust in nurses from the patients’ perspective could be related to factors previously reported. For example, some of the preconditions for the development of trust between patients and nurses were things like nurses’ levels of education and time employed; nurses’ availability and accessibility; their being adequately informed and communicated with respectfully; nurses’ technical or pedagogical competence; their experience and good bedside manner, continuity of service, and holistic approach to caring [52]. Factors that hindered the development of trust in nurse–patient relationships were things like lacking the necessary knowledge and skill to undertake nursing procedures; using difficult scientific language; not understanding patients’ needs; depersonalising the patient by not calling them by their names but by a room number or their diagnosis; avoiding nursing care activities; and keeping a distance from patients. Factors that were related to their job, such as lack of time, demanding workload, and absence of understanding [52], also undermined patients’ trust in nurses. Most of these factors were already identified as barriers to diabetes care in our study. Therefore, one could conclude that the nurse participants’ observations of a lack of trust from the patients’ perspectives is reasonable and that it is a cycle with factors affecting each other.

## 6. Conclusions

Significant results were found in relation to the barriers to diabetes inpatient care. Crucially, the findings demonstrate that all these factors can negatively affect the quality of care of inpatients with diabetes, and that most of these factors are not only related to diabetes care but generally to all patients who receive inpatient care. Interestingly, no participant reported any facilitators to his or her care, which further affected the negative perceptions of the care received. No other studies have reported on the inpatient diabetes care aspect, and the evidence generated in this study demonstrates an important strength of the diabetes care nurses provide, which may also help alleviate the concerns expressed by other healthcare professionals and groups of patients. Although beyond the scope of the current study, future studies should also evaluate other aspects of diabetes inpatient services, such as doctors’ provision of care or the role of other health professionals involved in diabetes care, in order to have additional data that would more comprehensively illustrate diabetes inpatient care, in general. Further research can be conducted on the factors affecting people with diabetes, such as the data that patients are more prone to trust doctors rather than nurses, which is opposed to other research evidence.

## Abbreviations

T1DM	Type 1 diabetes mellitus
T2DM	Type 2 Diabetes Mellitus
DSN	Diabetes Specialist Nurse
CoMeTS	Comparative Method for Themes Saturation

## References

[1] Gunns D., Leach M.J. (2020). An increased focus on stress for the management of blood glucose levels in type 1 diabetes: A case report. Aust. J. Herb. Naturopathic. Med..

[2] Divers J., Mayer-Davis E.J., Lawrence J.M., Isom S., Dabelea D., Dolan L., Imperatore G., Marcovina S., Pettitt D.J., Pihoker C. (2020). Trends in Incidence of Type 1 and Type 2 Diabetes Among Youths - Selected Counties and Indian Reservations, United States, 2002–2015. MMWR Morb. Mortal Wkly. Rep..

[3] Chen Y., Wang T., Liu X., Shankar R.R. (2019). Prevalence of type 1 and type 2 diabetes among US pediatric population in the MarketScan Multi-State Database, 2002 to 2016. Pediatr. Diabetes.

[4] Hare M.J., Shaw J.E. (2019). Inpatient diabetes care requires adequate support, not just HbA1c screening. Med. J. Aust..

[5] Donze J., Lipsitz S., Bates B.W., Schinipper J.L. (2013). Causes and patterns of readmissions in patients with common comorbidities: Retrospecitve cohort study. BMJ.

[6] Akiboye F., Adderley N.J., Martin J., Gokhale K., Rudge G.M., Marshall T.P., Rajendran R., Nirantharakumar K., Rayman G. (2020). Impact of the Diabetes Inpatient Care and Education (DICE) project on length of stay and mortality. Diabetic. Med..

[7] NHS Digital (2017). National Diabetes Inpatient Audit (NADiA). https://digital.nhs.uk/data-and-information/publications/statistical/national-diabetes-inpatient-audit/national-diabetes-inpatient-audit-nadia-2017.

[8] Meng Y.Y., Pickett M., Babey S.H., Davis A.C., Goldstein H. (2014). Diabetes Tied to a Third of California Hospital Stays, Driving Healthcare Costs Higher.

[9] Levy N., Dhatariya K. (2019). Pre-operative optimisation of the surgical patient with diagnosed and undiagnosed diabetes: A practical review. Anaesthesia.

[10] Ostling S., Wyckoff J., Ciarkowski S., Pai C., Choe H., Bahl V., Gianchandani R. (2017). The relationship between diabetes mellitus and 30-day readmission rates. Clin. Diabetes Endocrinol..

[11] Comino E.J., Harris M.F., Islam M.D., Tran D.T., Jalaludin B., Jorm L., Flack J., Haas M. (2015). Impact of diabetes on hospital admission and length of stay among a general population aged 45 year or more: A record linkage study. BMC Health Serv. Res..

[12] NICE (2015). Type 1 Diabetes in Adults: Diagnosis and Management Guideline. https://www.nice.org.uk/guidance/ng17.

[13] Tao B., Pietropaolo M., Atkinson M., Schatz D., Taylor D. (2010). Estimating the Cost of Type 1 Diabetes in the U.S.: A propensity Score Matching Methon. PLoS ONE.

[14] Watts S.A., Sood A. (2016). Diabetes nurse case management: Improving glucose control: 10 years of quality improvement follow-up data. Appl. Nurs. Res..

[15] Aliha J.M., Asgari M., Khayeri F., Ramazani M., Farajzadegan M., Javaheri J. (2013). Group education and nurse-telephone follow-up effects on blood glucose control and adherence to treatment in type 2 diabetes patients. Int. J. Prev. Med..

[16] Nikitara M., Constantinou C.S., Andreou E., Diomidous M. (2019). The Role of Nurses and the Facilitators and Barriers in Diabetes Care: A Mixed Methods Systematic Literature Review. Behav. Sci. (Basel).

[17] Breuker C., Macioce V., Mura T., Audurier Y., Boegner C., Jalabert A., Villiet M., Castet-Nicolas A., Avignon A., Sultan A. (2017). Medication errors at hospital admission and discharge in Type 1 and 2 diabetes. Diabetic Med..

[18] Taylor J.E., Campbell L.V., Zhang L., Greenfield J.R. (2018). High diabetes prevalence and insulin medication errors in hospital patients. Int. Med. J..

[19] Cardwell J., Hardy K., Ford N., O’Brien S. (2016). Assessment of diabetes knowledge in trained and untrained ward nurses before and after intensive specialist support. J. Diabetes Nurs..

[20] Williams I., Allen K., Plane G. (2019). Reports of rationing from the neglected realm of capital investment: Responses to resource constraint in the English National Health Service. Soc. Sci. Med..

[21] NHS Digital (2016). National Diabetes Inpatient Audit (NaDIA)—2015.

[22] Tabesh M., Magliano D.J., Koye D.N., Shaw J.E. (2018). The effect of nurse prescribers on glycaemic control in type 2 diabetes: A systematic review and meta-analysis. Int. J. Nurs. Stud..

[23] Grant A.K., Golden L. (2019). Technological Advancements in the Management of Type 2 Diabetes. Curr. Diabetes Rep..

[24] Sherr D., Lipman R.D. (2015). The Diabetes Educator and the Diabetes Self-management Education Engagement: The 2015 National Practice Survey. Diabetes Educ..

[25] Riordan F., McHugh S.M., Murphy K., Barrett J., Kearney P.M. (2017). The role of nurse specialist of integrated diabetes care: A cross sectional survey of diabetes nurse specialist services. BMJ Open.

[26] Smith J.A., Osborn M., Smith J. (2008). Interpretative Phenomenological Analysis. Qualitative Psychology: A Practical Guide to Research Methods.

[27] Smith. J.A., Harre R., van Langenhove L. (1995). Ideography and the Case Study. Rethinking Psychology.

[28] Constantinou C.S., Georgiou M., Perdikogianni M. (2017). A comparative method for themes saturation (CoMeTS) in qualitative interviews. Qual. Res..

[29] Vasileiou K., Barnett J., Thorpe S., Young T. (2018). Characterising and justifying sample size sufficiency in interview-based studies: Systematic analysis of qualitative health research over a 15-year period. BMC Med. Res. Methodol..

[30] Raaijmakers L.G.M., Hamers F.J.M., Martens M.K., Bagchus C., de Vries N.K., Kremers S.P.J. (2013). Perceived facilitators and barriers in diabetes care: A qualitative study among healthcare professionals in the Netherlands. BMC Fam. Prac..

[31] O'Connor R., Mannix M., Mullen J., Powys L., Mannion M., Nolan H.A., Kearney E., Cullen W., Griffin M., Saunders J. (2013). Structured care of diabetes in general practice: A qualitative study of the barriers and facilitators. Irish Med. J..

[32] Roopnarinesingh N., Brennan N., Khan C., Ladenson P.W., Hill-Briggs F. (2015). Barriers to optimal diabetes care in Trinidad and Tobago: A Healthcare Professionals’ perspective. BMC Health Serv. Res..

[33] Rivaz M., Momennasab M., Yektatalab S., Ebadi A. (2017). Adequate Resources as Essential Component in the Nursing Practice Environment: A Qualitative Study. J. Clin. Diagn. Res..

[34] Blackman I., Henderson J., Willis E., Hamilton P., Toffoli L., Verrall C., Abery E., Harvey C. (2015). Factors influencing why nursing care is missed. J. Clin. Nurs..

[35] Hunt P.A., Denieffe S., Gooney M. (2017). Burnout and its relationship to empathy in nursing: A review of the literature. J. Res. Nurs..

[36] Jeffrey D. (2016). Empathy, sympathy and compassion in healthcare: Is there a problem? Is there a difference? Does it matter?. J. R. Soc. Med..

[37] Wilkinson H., Whittington R., Perry L., Eames C. (2017). Examining the relationship between burnout and empathy in healthcare professionals: A systematic review. Burnout Res..

[38] Duarte J., Pinto-Gouveia J., Cruz B. (2016). Relationships between nurses' empathy, self-compassion and dimensions of professional quality of life: A cross-sectional study. Int. J. Nurs. Stud..

[39] Santos E.I., Alves Y.R., Gomes A.M.T., Ramos R.S., Silva A.C.S.S., Santo C.C.E. (2015). Representações sociais da autonomia profissional do enfermeiro para profissionais de saúde não-enfermeiros. Rev. Enferm. UERJ.

[40] Baykara Z.G., Sahinoglu S. (2014). An evaluation of nurse’s professional autonomy in Turkey. Nurs. Ethics..

[41] Kramer M., Schmalenberg C.E. (2003). Magnet hospital staff nurses describe clinical autonomy. Nurs. Outlook.

[42] Traynor M., Boland M., Buus N. (2010). Autonomy, evidence, and intuition: Nurses and decision-making. J. Adv. Nurs..

[43] Wade G.H. (1999). Professional nurse autonomy: Concept analysis and application to nursing education. J. Adv. Nurs..

[44] Rao A.D., Kumar A., McHugh M. (2018). Better Nurse Autonomy Decreases the Odds of 30-Day Mortality and Failure to Rescue. J. Nurs. Scholarsh.

[45] Kramer M., Maguire P., Schmalenberg C.E. (2006). Excellence through evidence: The what, when, and where of clinical autonomy. J. Nurs. Admin..

[46] Varjus S., Leino-Kilpi H., Suominen T. (2011). Professional autonomy of nurses in hospital settings—A review of the literature. Scand. J. Caring Sci..

[47] Aiken L.H., Clarke S.P., Sloane D.M., Lake E.T., Cheney T. (2008). Effects of hospital care environment on patient mortality and nurse outcomes. J. Nurs. Admin..

[48] Rafferty A., Ball J., Aiken L.H. (2001). Are teamwork and professional autonomy compatible, and do they result in improved hospital care?. Qual. Health Care.

[49] Bonfada M.S., Pinno C., Camponogara S. (2018). Potentialities and limits of nursing autonomy in a hospital environment. J. Nurs..

[50] Loizou C., Phellas C. (2009). Social Dimensions of diabetes mellitus in Cyprus. Society & Health: Psycho-Social Aspects in the Greek & Cypriot Region.

[51] Huang E.C.-H., Pu C., Chou Y.-J., Huang N. (2018). Public Trust in Physicians--Healthcare Commodification as a Possible Deteriorating Factor: Cross-sectional Analysis of 23 Countries. Inquiry.

[52] Dinç L., Gastmans C. (2013). Trust in nurse-patient relationships: A literature review. Nurs. Ethics..

